# Assessing Visceral Obesity and Abdominal Adipose Tissue Distribution in Healthy Populations Based on Computed Tomography: A Large Multicenter Cross-Sectional Study

**DOI:** 10.3389/fnut.2022.871697

**Published:** 2022-04-25

**Authors:** Ming Kong, Manman Xu, Ying Zhou, Nan Geng, Ning Lin, Wenyan Song, Shanshan Li, Yuetong Piao, Zuoqing Han, Rong Guo, Chao Yang, Nan Luo, Zhong Wang, Lei Ma, Quanxiao Xu, Lili Wang, Wanchun Qiu, Junfeng Li, Daimeng Shi, Eddie C. Cheung, Rongkuan Li, Yu Chen, Zhongping Duan

**Affiliations:** ^1^Beijing Municipal Key Laboratory of Liver Failure and Artificial Liver Treatment Research, Fourth Department of Liver Disease, Beijing Youan Hospital, Capital Medical University, Beijing, China; ^2^Postgraduate Training Base of Jinzhou Medical University, Department of Gastroenterology and Hepatology, The Chinese People's Liberation Army Rocket Force Characteristic Medical Center, Beijing, China; ^3^Department of Radiology, Beijing Youan Hospital, Capital Medical University, Beijing, China; ^4^Department of Infection, Dalian Medical University, Dalian, China; ^5^Department of Radiology, The Second Hospital of Dalian Medical University, Dalian, China; ^6^Department of Infection, The Second Hospital of Dalian Medical University, Dalian, China; ^7^Diagnosis and Treatment Center of Hepatobiliary Diseases, Nanyang First People's Hospital, Nanyang, China; ^8^Department of Radiology, The First Hospital of Lanzhou University, Lanzhou, China; ^9^Department of Infection, The First Clinical Medical School of Lanzhou University, Lanzhou, China; ^10^Department of Infectious Diseases, Institute of Infectious Diseases, The First Hospital of Lanzhou University, Lanzhou, China; ^11^Division of Gastroenterology, School of Medicine, University of California, Davis, Davis, CA, United States; ^12^Center for Digestive Disease, The Seventh Affiliated Hospital, Sun Yat-sen University, Shenzhen, China

**Keywords:** visceral adipose tissue area, subcutaneous adipose tissue area, computed tomography, visceral obesity, normal body mass index

## Abstract

**Objective:**

Abdominal adipose is closely related to many endocrine and metabolic diseases. The aim of this study was to analyze the distribution of abdominal adipose tissue in a healthy population in northern China determined by abdominal computed tomography (CT).

**Methods:**

Data for this study were obtained from a multicenter, retrospective, cross-sectional study that collected abdominal CT scans of 1787 healthy individuals from 4 representative cities in northern China. Areas of visceral adipose tissue (VATA) and subcutaneous adipose tissue (SATA) were obtained by measuring CT images at the level of the 3rd lumbar vertebra. Visceral adipose tissue index (VATI) and subcutaneous adipose index (SATI) were obtained by normalizing the square of height to analyze the distribution of the above indexes and visceral obesity among different body mass index (BMI), gender and age.

**Results:**

The mean age of this healthy population was 45.3 ± 15.2 years and the mean BMI was 23.5 ± 3.2 kg/m^2^, with 902 men and 885 women. Compared with women, men had a significantly higher median VATA (120.9 vs. 67.2 cm^2^), VATI (39.1 vs. 25.6 cm^2^/m^2^) and a significantly higher percentage of visceral adiposity (VATA ≥ 100 cm^2^) (60.8 vs. 30.4%), while women had significantly higher SATA (116.9 vs. 146.7 cm^2^) and SATI (38.8 vs. 55.8 cm^2^/m^2^) than men. Whether men or women, VATI was positively correlated with age. Interestingly, SATI was weakly positively correlated with age in women, while SATI was weakly negatively correlated with age in men. In persons with a normal BMI, the proportion of visceral adiposity increases with age, whereas in men with a normal BMI, the proportion of visceral adiposity decreases after the age of 60 years but remains >50%.

**Conclusions:**

The distribution of abdominal visceral and subcutaneous adipose tissue parameters measured by CT differed among gender, age, and BMI. Even men and women with normal BMI have a high proportion of visceral obesity.

## Introduction

Obesity is caused by an imbalance between energy intake and expenditure and is characterized by the accumulation of adipose tissue in both visceral and subcutaneous depots (1). Obesity can also alter the endocrine and metabolic functions of adipose tissue and is a risk factor for many metabolic diseases (2). The regional distribution of adipose tissue has been shown to be a stronger predictor of health risk than overall excessive adiposity (3). Excess of visceral adipose tissue facilitates high doses of adipokines in the portal vein to the liver and other body tissues, resulting in serious effects such as diabetes, non-alcoholic fatty liver diseases, kidney disease, cancer and other health problems (4).Studies have shown that visceral obesity is also associated with increased COVID-19 severity (5). A large prospective multiethnic cohort study demonstrated that visceral adipose tissue (VAT), but not body mass index (BMI) and subcutaneous adipose tissue (SAT), was significantly associated with increased risk of cardiovascular disease (6). It has also been reported that visceral obesity as measured by computed tomography (CT) is positively associated with the development of colonic diverticulosis, even at normal body weight (7). Therefore, we should pay attention to the health problems brought by the occurrence risk of visceral obesity.

Computed tomography (CT) imaging is a method to accurately quantify the regional distribution of abdominal adipose tissue parameters and is considered the gold standard for body composition assessment (8). It has been reported that different races, eating habits, physical activity and other factors may contribute to different body composition (9). In northern China, there has been little research on the potential demographic heterogeneity of the regional distribution of abdominal adipose and the risk of visceral obesity with different BMI. Therefore, the aim of our study was to analyze the distribution of adipose tissue at the level of the third lumbar vertebra and the proportion of visceral adiposity in a healthy population of different gender, age, and BMI.

## Methods

### Study Population

In this multicenter retrospective cross-sectional study (10), we collected 1,787 healthy adults from January 2016 to March 2021 from tertiary hospitals in 4 representative cities in northern China (showed in Supplementary Figure 1). Individuals who met the following criteria were included: (1) age over 20 years; (2) having an analysable abdominal CT examination (including third lumbar vertebrae (L3) level) and (3) BMI ≥ 18.5 kg/m^2^. Individuals were excluded from the study if they had (1) malignancy; (2) various chronic diseases, such as cardio-cerebrovascular diseases, chronic obstructive pulmonary disease, chronic hepatitis, and chronic renal insufficiency; (3) various serious diseases, such as liver failure, respiratory and circulatory failure, renal failure, and severe acute pancreatitis; (4) endocrine and metabolic syndrome, such as thyroid dysfunction and diabetes mellitus; (5) autoimmune diseases presently taking glucocorticoids; and (6) mental illness. Data collected included sex, age, height, weight, BMI and CT scan. World health organization (WHO) (11) standards were used to categorize BMI as follows: normal/healthy weight (18.5–25.0 kg/m^2^), overweight (25.0–30.0 kg/m^2^), and obesity (≥30.0 kg/m^2^). Diagnostic criteria for visceral obesity used previously reported visceral adipose tissue area ≥ 100 cm^2^ at L3 level in both men and women (12). This criterion was used as a cutoff to define myopenia obesity in Asian populations and to diagnose visceral obesity.

The retrospective study procedures were conducted in accordance with the Declaration of Helsinki and approved by the Ethics Committee of Beijing Youan Hospital (LL-2021-018-K). We only obtained body composition data and relevant clinical information, and not involved the privacy of participants. The ethics committee did not require participants to sign informed consent.

### Abdominal Adipose Tissue Assessment

**Figure 1:**
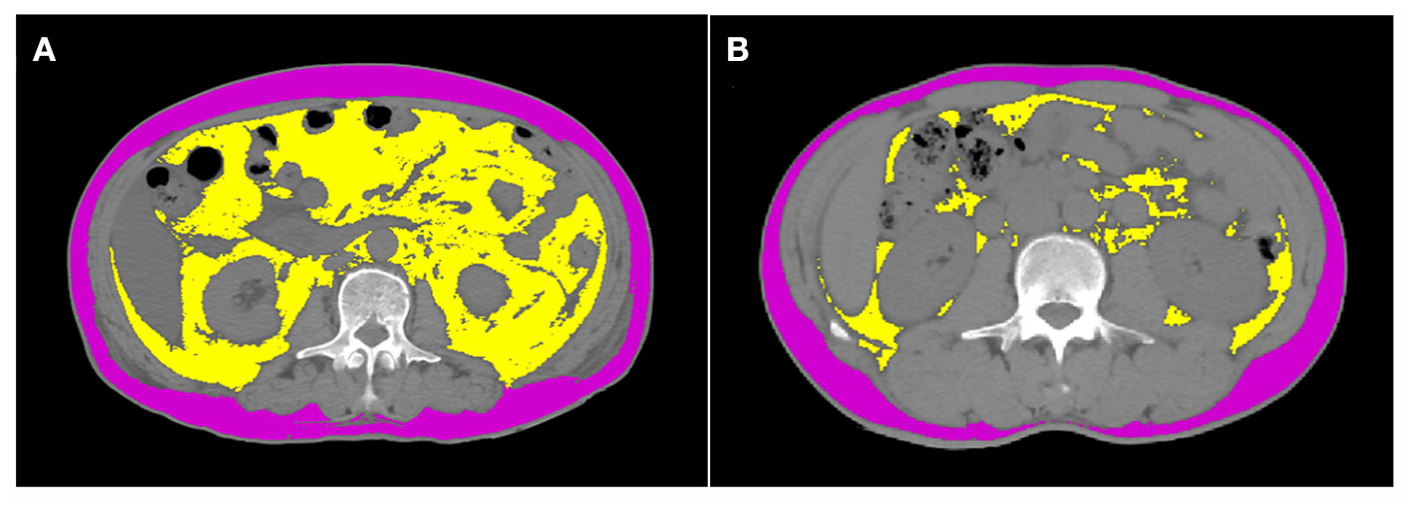
Computed tomograph scan shows the body composition of normal BMI individuals with and without visceral obesity. **(A)** represents a normal BMI with visceral obesity, with a BMI of 23.2 kg/m^2^ and visceral adipose tissue of 207 cm^2^. **(B)** represents a normal BMI without visceral obesity, with a BMI of 23.2 kg/m^2^ and visceral adipose tissue of 23.9 cm^2^. Visceral adipose tissue area is highlighted in yellow and the subcutaneous adipose tissue area is purple.

In this study, included healthy people all underwent abdominal CT scan, CT units used and detailed technical parameters showed in **Supplementary Table 1**. We derived cross-sectional CT images of the mid-third lumbar vertebrae (L3) from the PACS system in the radiology department and studied them in dicom format. Adipose tissue parameters were quantified using SliceOmatic (V5.0, Tomovision, Magog, Canada) software. Tissue segmentation was performed using Hounsfield unit thresholds of −150 to −50 for visceral adipose tissue and −190 to −30 for subcutaneous adipose tissue (13) (Figure 1). Visceral adipose tissue area (VATA) is highlighted in yellow and the subcutaneous adipose tissue area is purple. Notably, adipose in the kidney, liver and intestines should not be included in VAT, despite having the same density values as VAT. The cross-sectional of VATA and SATA were automatically calculated, and then normalized for height squared to obtain the visceral adipose tissue index (VATI, cm^2^/m^2^) and subcutaneous adipose tissue index (SATI, cm^2^/m^2^). L3-total adipose tissue area (L3-TATA) was the sum of L3-VATA and L3-SATA.

### Statistical Analysis

We used R×64 4.0.3 (http://www.r-project.org/) and Origin 9.0 (origin lab, Northampton, MA, USA) to analyze our data and make diagrams. *P* < 0.05 was considered statistically significant. Categorical variables were expressed as number (percentages) and were compared by the chi-square test. Continuous data were shown as the median (interquartile range). Comparison of continuous variables between two groups was performed by either the Mann-Whitney U test. Correlations between adipose tissue indices (VATI, SATI, and TATI) and age and BMI were visualized by scatter plots and assessed using Pearson correlation coefficients.

## Results

### Baseline Characteristics of Study Population

A total of 1787 healthy people were included in the sample, including 902 men (50.5%) and 885 women (49.5%). The mean age of the sample was 45.3 ± 15.2 years (range from 20 to 88 years) and the mean BMI was 23.5 ± 3.2 kg/m^2^ (range from 18.5 to 38.7 kg/m^2^). The normal weight, overweight, and obese groups in the study population were 1,284, 434, and 69 individuals, respectively. Table 1 shows the comparison of characteristics and abdominal adipose parameters among different genders of the study population. The age between the two groups were not statistically significant (44.0 vs. 46.0 years, *P* = 0.310), but height, weight and BMI were significantly higher in men than in women (*P* < 0.001). For abdominal adipose parameter analysis, men had higher median VATA (120.9 vs. 67.2 cm^2^, *P* < 0.001), VATI (39.1 vs. 25.6 cm^2^/m^2^, *P* < 0.001) and TATA (244.0 vs. 221.0 cm^2^, *P* = 0.004) than women. Compared to men, women had more median SATA (116.9 vs. 146.7 cm^2^, *P* < 0.001), SATI (38.8 vs. 55.8 cm^2^/m^2^, *P* < 0.001), and TATI (81.2 vs. 83.7 cm^2^/m^2^, *P* = 0.003). The percentage of visceral obesity was significantly higher in men than in women (60.8 vs. 30.4%, *P* < 0.001).

**Table 1:** Baseline characteristics of the study population according to sex.

**Variables**	**Total (***n*** = 1,787)**	**Male (***n*** = 902)**	**Female (***n*** = 885)**	***P*** **-value**
Age, years	45.0 (25.0)	44.0 (24.0)	46.0 (24.5)	0.310
Height, m	1.68 (0.13)	1.74 (0.08)	1.62 (0.05)	<0.001
Weight, kg	65.0 (15.0)	70.0 (14.0)	60.0 (10.0)	<0.001
BMI, kg/m^2^	23.1 (4.1)	23.4 (4.1)	22.9 (4.3)	<0.001
Normal weight, *n* (%)	1284 (71.9%)	630 (70.0%)	654 (73.9)	
Overweight, *n* (%)	434 (24.3%)	237 (26.3%)	197 (22.3%)	
Obese, *n* (%)	69 (3.8%)	35 (3.9%)	34 (3.8%)	
L3-VATA, cm^2^	90.6 (100.2)	120.9 (104.9)	67.2 (79.8)	<0.001
L3-VATI, cm^2^/m^2^	32.2 (34.5)	39.1 (34.8)	25.6 (29.8)	<0.001
L3-SATA, cm^2^	129.9 (79.9)	116.9 (66.1)	146.7 (86.5)	<0.001
L3-SATI, cm^2^/m^2^	45.6 (29.1)	38.8 (21.6)	55.8 (33.4)	<0.001
L3-TATA, cm^2^	233.1 (155.9)	244.0 (154.1)	221.0 (148.9)	0.004
L3-TATI, cm^2^/m^2^	82.1 (53.4)	81.2 (51.6)	83.7 (57.2)	0.003
Visceral obesity (VAT ≥ 100 cm^2^), %	817 (45.7%)	548 (60.8%)	269 (30.4%)	<0.001

*Data are presented as median (interquartile range) for non-normally distributed continuous variables*.

*Categorical variables are shown as number (percentages %)*.

### Correlation of Adipose Tissue Parameters With Age and BMI

Table 2 shows the comparison of abdominal adipose parameters across gender and age. Stratified by sex and age (20–29, 30–39, 40–49, 50–59, ≥60 years), our data showed that abdominal adipose parameters presented different trends of change in men and women. In men, VATA and VATI gradually increased before the age of 60 and decreased after the age of 60; While the SATA and SATI showed an increasing trend with age before 40 years old and a decreasing trend over 40 years old. While in women, all adipose parameters (VATA, VATI, SATA, and SATI) increased with increasing age. Correlation analysis showed that both VATI and TATI were moderately and positively correlated with age (r = 0.522, *P* < 0.001; r = 0.362, *P* < 0.001) in women, and the correlation was higher than that in men (r = 0.254, *P* < 0.001; r = 0.125, *P* < 0.001).

**Table 2:** Distribution of body composition by sex and age.

**Sex**	**Male (*****n*** **= 902)**					**Female (*****n*** **= 885)**				
Age, years	20–29 (*n* = 185)	30–39 (*n* = 178)	40–49 (*n* = 180)	50–59 (*n* = 180)	≥60 (*n* = 179)	20–29 (*n* = 169)	30–39 (*n* = 168)	40–49 (*n* = 182)	50–59 (*n* = 184)	≥60 (*n* = 182)
**Variables**
BMI, kg/m^2^	21.8 (3.7)	24.2 (4.5)	23.9 (4.4)	23.7 (3.9)	22.8 (3.3)	20.9 (3.8)	22.2 (4.5)	22.9 (3.2)	23.4 (4.2)	23.9 (4.2)
Normal weight, *n* (%)	149 (80.5%)	106 (59.6%)	111 (61.7%)	122 (67.8%)	142 (79.3%)	143 (84.6%)	133 (79.2%)	142 (78.0%)	119 (64.7%)	117 (64.3%)
Overweight, *n* (%)	30 (16.2%)	57 (32.0%)	60 (33.3%)	54 (30.0%)	36 (20.1%)	20 (11.8%)	24 (14.3%)	38 (20.9%)	58 (31.5%)	57 (31.3%)
Obese, *n* (%)	6 (3.2%)	15 (8.4%)	9 (5.0%)	4 (2.2%)	1 (0.6%)	6 (3.6%)	11 (6.5%)	2 (1.1%)	7 (3.8%)	8 (4.4%)
L3-VATA, cm^2^	60.3 (91.5)	126.2 (84.6)	131.4 (100.2)	147.2 (93.7)	122 (107.9)	28.3 (49.7)	49.4 (57.3)	65.5 (55.3)	81.3 (66.7)	120.2 (74.9)
L3-VATI, cm^2^/m^2^	19.2 (29.0)	41.1(27.5)	44.9 (31.8)	48.2 (32.2)	42.6 (35.1)	10.5 (19.6)	17.6 (20.9)	25.1 (22.0)	31.7 (23.2)	46.6 (28.7)
L3-SATA, cm^2^	109.4 (103.7)	132.4 (68.6)	127.1 (64.1)	116.3 (59.9)	108.6 (49.6)	127.2 (91.9)	134.7 (84.8)	146.9 (64.8)	158.8 (83.7)	156.7 (85.4)
L3-SATI, cm^2^/m^2^	34.9 (33.1)	43.0 (20.1)	41.8 (19.9)	38.8 (18.9)	36.4 (16.8)	47.6 (34.6)	50.0 (32.2)	56.8 (26.8)	59.6 (32)	62.1 (32.9)
L3-TATA, cm^2^	172.3 (190.8)	263.9 (119.8)	260.9 (142.6)	263.5 (137.8)	231.7 (140.2)	157.4 (132.9)	183.7 (146.2)	216.8 (104.0)	243.0 (133.8)	288.2 (155.6)
L3-TATI, cm^2^/m^2^	56.5 (60.7)	86.8 (144.6)	86.8 (50.8)	88.3 (48)	78.5 (50.5)	57.7 (50.8)	69.7 (55.3)	81.9 (40.8)	91.6 (47.1)	111.2 (58.4)

*Data are presented as median (interquartile range) for non-normally distributed continuous variables*.

*Categorical variables are shown as number (percentages %)*.

**Figure 2:**
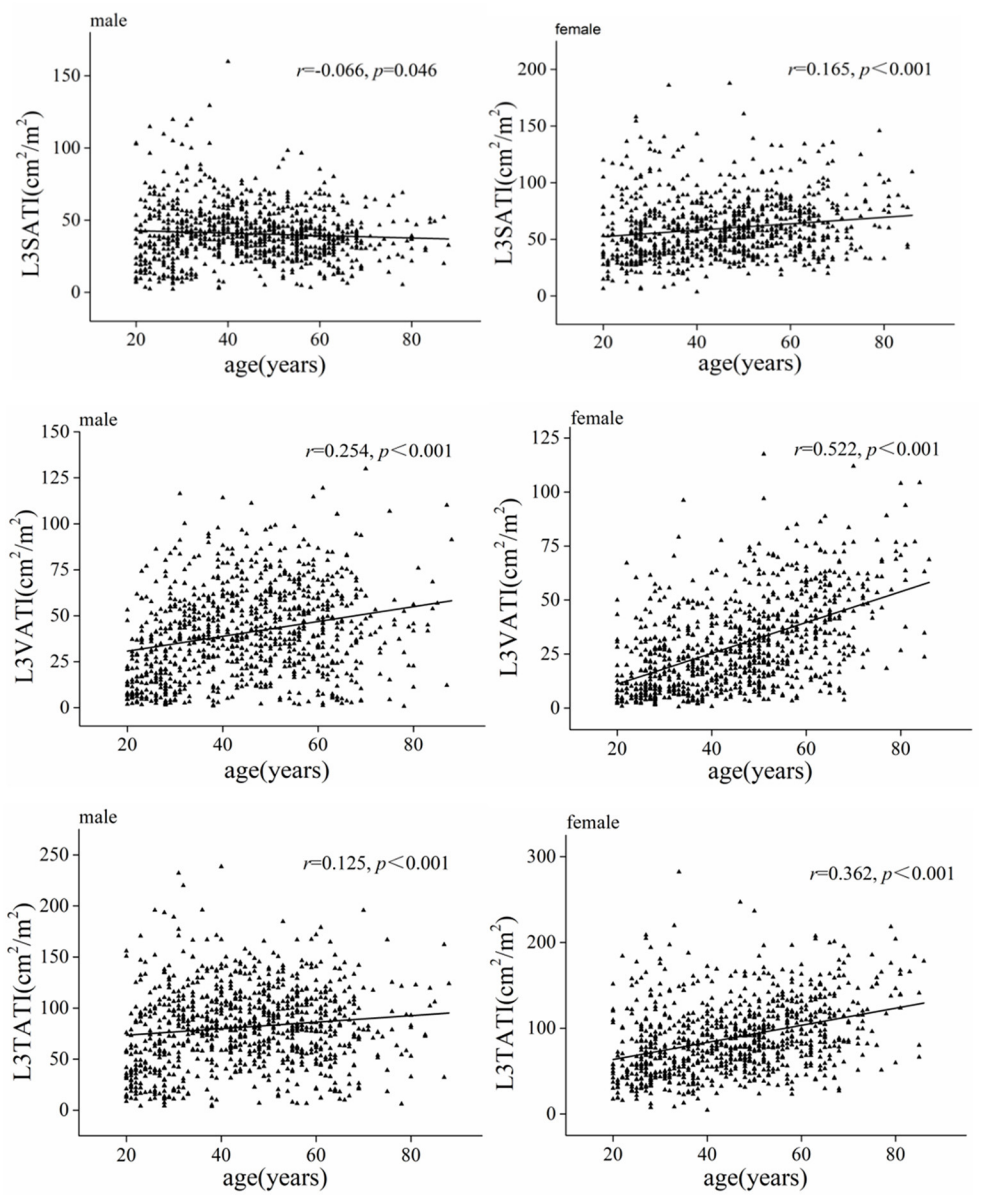
Scatter graph depicting correlations between adipose tissue index (VATI, SATI, and TATI) and age classified according to sex.

In addition, a weak positive correlation was observed between SATI and age in women (r = 0.165, *P* < 0.001), while a weak negative correlation was observed between SATI and age in men (r = −0.066, *P* = 0.046) (Figure 2).

**Figure 3:**
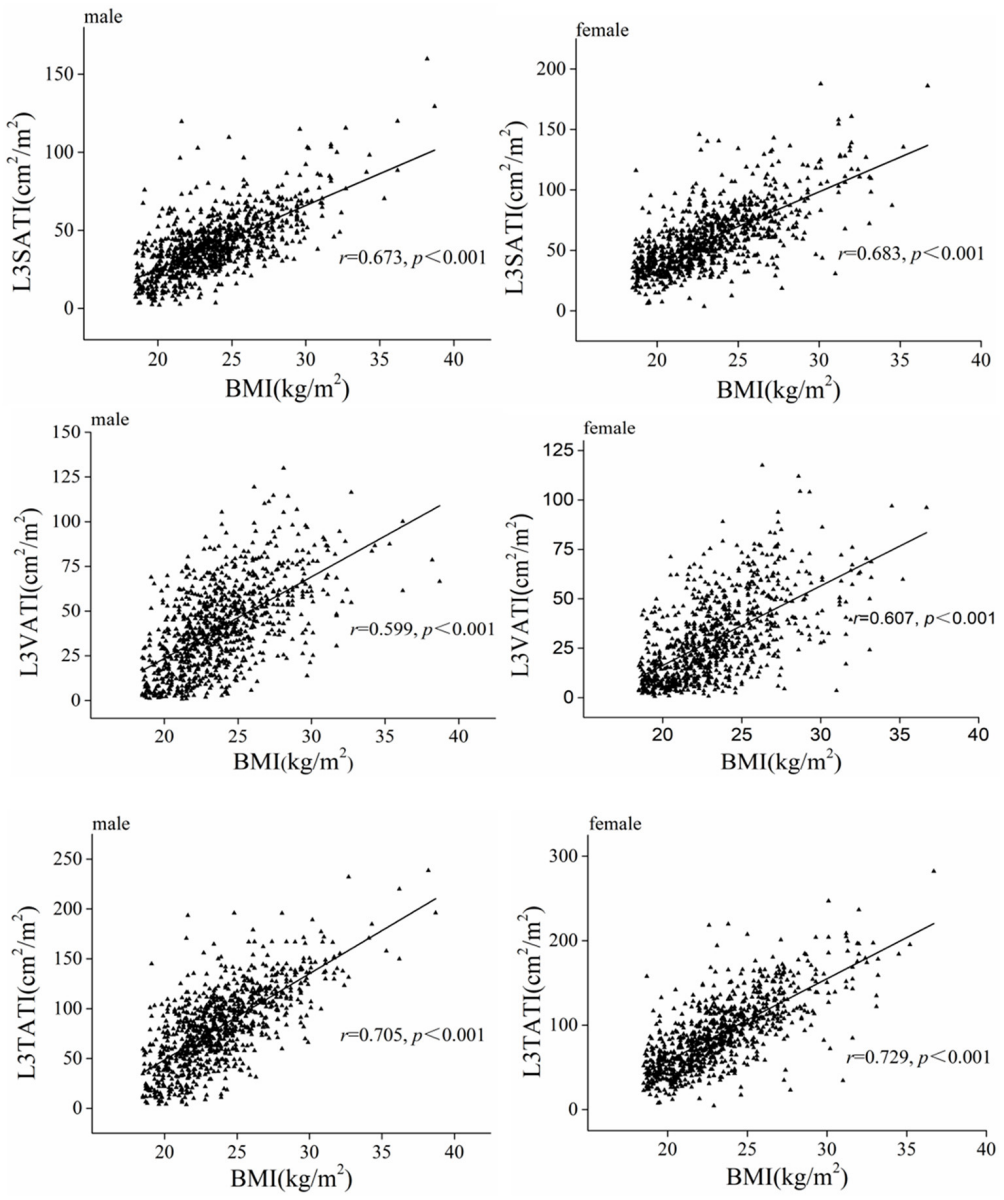
Scatter graph depicting correlations between adipose tissue index (VATI, SATI, and TATI) and BMI classified according to sex.

Figure 3 shows the correlations between BMI and abdominal adipose parameters in different genders, and the results showed that adipose parameters (VATI, SATI and TATI) were significantly positively correlated with BMI in both men and women (*P* < 0.001). Similarly, the adipose parameters (VATA, VATI, SATA, SATI, TATA, and TATI) increased gradually in normal weight, overweight and obese people (showed in Supplementary Table 2).

### Distribution of Visceral Obesity Across Sex, Age, and BMI

**Figure 4:**
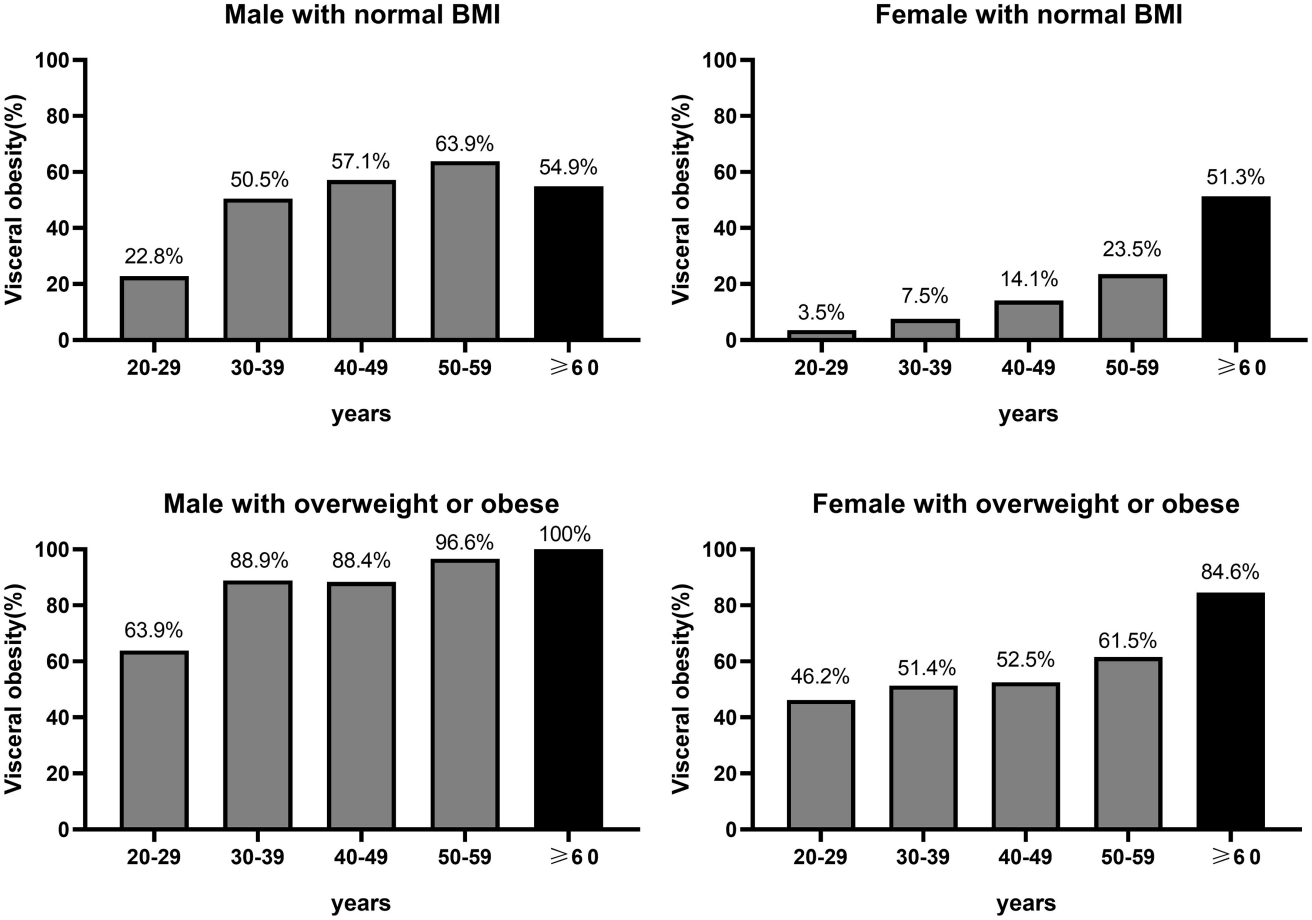
Proportion of visceral adiposity in different age strata in people with different BMI.

Among women with normal BMI, the proportion of visceral obesity increased progressively with age, with 3.5, 7.5, 14.1, 23.5, and 51.3% in 20–29, 30–39, 40–49, 50–59, and ≥60 years (Figure 4). Whereas among men with normal BMI, the proportion of visceral obesity increased gradually before the age of 60 years, but there was a slight decrease in the percentage of visceral obesity in older men (≥60 years), with 22.8, 50.5, 57.1, 63.9, and 54.9% in 20–29, 30–39, 40–49, 50–59, and ≥60 years, respectively (Figure 4). It was noted that the percentage of visceral obesity in both older men and older women with a normal BMI was more than 50%. Among overweight or obese (BMI ≥ 25 kg/m^2^) men and women, the proportion of visceral obesity increased progressively with age, with corresponding proportions of 46.2, 51.4, 52.5, 61.5, and 84.6% among women aged 20–29, 30–39, 40–49, 50–59, and ≥60 years, respectively, and 63.9, 88.9, 88.4, 96.6, and 100% among men, respectively (Figure 4).

## Discussion

Adipose tissue, as an important part of body composition, plays an important role in energy regulation. It also plays an important role in regulating lipid metabolism, glucose metabolism, insulin sensitivity, angiogenesis, appetite and inflammation by producing adipokines (4). Analysis of body composition parameters is a new method that can be easily obtained from CT images. There are few studies on the percentage of normal BMI with visceral obesity in healthy people in northern China. To this end, we collected 1,787 healthy individuals from 4 representative cities in northern China who underwent abdominal CT for analysis of abdominal adipose distribution. We found differences in the distribution of abdominal adipose tissue by gender, age and BMI. The proportion of visceral obesity was significantly higher in men than in women, regardless of age. Interestingly, we found that the percentage of visceral obesity was high in both men and women with normal BMI, and the percentage of visceral obesity in older people was even more than 50%.

It is well known that the obesity epidemic constitutes a major public health problem, as obesity increases the risk of many diseases (14). The study showed that the regional distribution of adipose tissue was a stronger predictor of health risk than overall obesity (3). Abdominal adipose consists mainly of two types of adipose tissue, visceral and subcutaneous adipose. For gender analysis, our data showed that there were gender differences in the distribution of VATA and SATA. VATA and VATI in males were higher than those in females, while SATA and SATI in females were higher than those in males. Sexual dimorphism in the distribution of adipose tissue may be due to different sex hormones (15). From an evolutionary point of view, it may also be related to different reproductive strategies (16). Studies have shown that men have a higher risk of metabolic damage and related diseases (insulin resistance and type 2 diabetes) than women (17). VATA is closely associated with insulin resistance and type 2 diabetes, cardiovascular disease, hypertension, cancer, sleep apnea, and metabolic syndrome (18). Therefore, men may have a higher risk of having more visceral fat than women. It has also been reported that reduced SATA is associated with metabolic syndrome in women, but increased SATA has an adverse effect on metabolic syndrome in men (19). Low SATA in women was also associated with increased mortality in cirrhosis patients (20). Therefore, it is more advantageous for women to have a high SATA and men to have a low SATA. The present findings corroborate that men have a higher risk of developing metabolic diseases than women.

Our data showed that VATI was positively correlated with increasing age for both male and female. VATA in women is more susceptible to age. It has been reported that the prevalence of cardiovascular disease increases with age, and there are more risk factors for cardiovascular disease in the elderly than in the young (18). This may be due to the fact that most elder people have higher visceral fat than younger people.

The difference between men and women in VATA accumulation decreased with the increase of age (21). It has also been reported that premenopausal women have a lower risk of cardiac metabolic disease than men of the same age and BMI, but the risk of metabolic disease is similar in postmenopausal women and men (22). Our data also illustrated this phenomenon. The median VATA of premenopausal women was lower than that of men of the same age and BMI, and when older than 60 years, the median VAT of men and women was similar (122.0 vs. 120.2 cm^2^). For subcutaneous adipose tissue analysis, our data showed that the correlation between subcutaneous adipose tissue and age was reversed between men and women. A multicenter study has similar results, but the underlying mechanism for this phenomenon needs further investigation (23).

Visceral obesity has become a more reliable indicator of obesity than BMI in Asian populations (24). Studies have shown that visceral obesity may be a more useful clinical predictor than BMI in predicting the outcome of laparoscopic colorectal cancer surgery (25). A literature review has reported that high VATA is also superior to BMI in assessing the severity and prognosis of acute pancreatitis (26). Studies have also shown that subjects with visceral obesity but without overall obesity (VATA ≥ 100 cm^2^ plus BMI < 25 kg/m^2^) have more metabolic risk factors than overall obese subjects without visceral obesity (VATA < 100 cm^2^ plus BMI ≥ 25 kg/m^2^) (27). Therefore, not all obese people develop diabetes and cardiovascular disease, and these diseases seem to be associated with visceral obesity even in lean people (28). Our results showed that the percentage of visceral obesity was high in people with normal BMI, and the percentage of visceral obesity in older people was even more than 50%. This prompts us that visceral obesity needs to be managed more carefully in healthy people with normal BMI, otherwise the risk of many underlying diseases may be overlooked. It has been reported that quitting smoking and controlling healthy drinking habits were essential to prevent visceral obesity and related complications (29, 30). It has also been reported that combined aerobic exercise and resistance exercise may reduce the occurrence of sarcopenia obesity (31). Reducing visceral obesity in general may reduce the risk of coronary heart disease and metabolic disease in different populations (27).

Our study was not without its limitations. First, this study was designed as a retrospective study, which may have selective bias. Second, risk factors associated with visceral obesity, such as smoking, alcohol consumption, physical exercise and dietary habits, were not included in our study. Therefore, we can conduct a large sample, randomized, prospective study to further validate our results.

In this study, we found that the distribution of abdominal adipose tissue parameters measured by CT differed among sex, age and BMI. Interestingly, we found that the percentage of visceral obesity was high in both men and women with normal BMI, and the percentage of visceral obesity in older people was even more than 50%. Clinical and health guidelines generally do not recommend the assessment and management of normal-weight individuals with visceral obesity (32). The results of the present study suggested that we pay more attention to the problem of visceral obesity in normal weight people, and exercise and maintaining a healthy lifestyle were especially important for normal weight healthy people with visceral obesity.

## Data Availability Statement

The original contributions presented in the study are included in the article/Supplementary Material, further inquiries can be directed to the corresponding author/s.

## Ethics Statement

The studies involving human participants were reviewed and approved by the Ethics Committee of Beijing Youan Hospital (LL-2021-018-K). Written informed consent for participation was not required for this study in accordance with the national legislation and the institutional requirements.

## Author Contributions

ZD, YC, and MK conceived and designed the study. YC and ZD obtained funding. YZ, MX, NG, NLi, WS, SL, YP, ZH, RG, NLu, ZW, LM, and QX collected the data of manuscript. WS, CY, WQ, and LW operated software. MX and YZ performed statistical analysis and drafted the manuscript. YC, ZD, MK, JL, DS, RL, and EC revised the manuscript. All authors read and approved the final version of the article.

## Funding

This study was supported by Beijing Advanced Innovation Center for Big Data-Based Precision Medicine (PXM2021_014226_000026); the National 13th 5-Year Plan for Hepatitis Research (2017ZX10203201-007); Capital's Funds for Health Improvement and Research (2021-1G-2181).

## Conflict of Interest

The authors declare that the research was conducted in the absence of any commercial or financial relationships that could be construed as a potential conflict of interest.

## Publisher's Note

All claims expressed in this article are solely those of the authors and do not necessarily represent those of their affiliated organizations, or those of the publisher, the editors and the reviewers. Any product that may be evaluated in this article, or claim that may be made by its manufacturer, is not guaranteed or endorsed by the publisher.

## Supplementary Material

Supplementary Figure 1 Four representative cities in northern China.

Supplementary Table 1 CT units used and detailed technical parameters.

Supplementary Table 2 Distribution of body composition by sex and BMI.

## Abbreviations

VATA: visceral adipose tissue area
VATI: visceral adipose tissue index
SATA: subcutaneous adipose tissue area
SATI: subcutaneous adipose tissue index
TATA: total adipose tissue area
TATI: total adipose tissue index
BMI: body mass index
HU: Hounsfield Units
CT: Computed tomography.
