# Supplementary figures and images for: Assessing Visceral Obesity and Abdominal Adipose Tissue Distribution in Healthy Populations Based on Computed Tomography: A Large Multicenter Cross-Sectional Study
Source: Front Nutr. 2022 Apr 25;9:871697. doi: 10.3389/fnut.2022.871697 (PMC9082940; doi:10.3389/fnut.2022.871697)

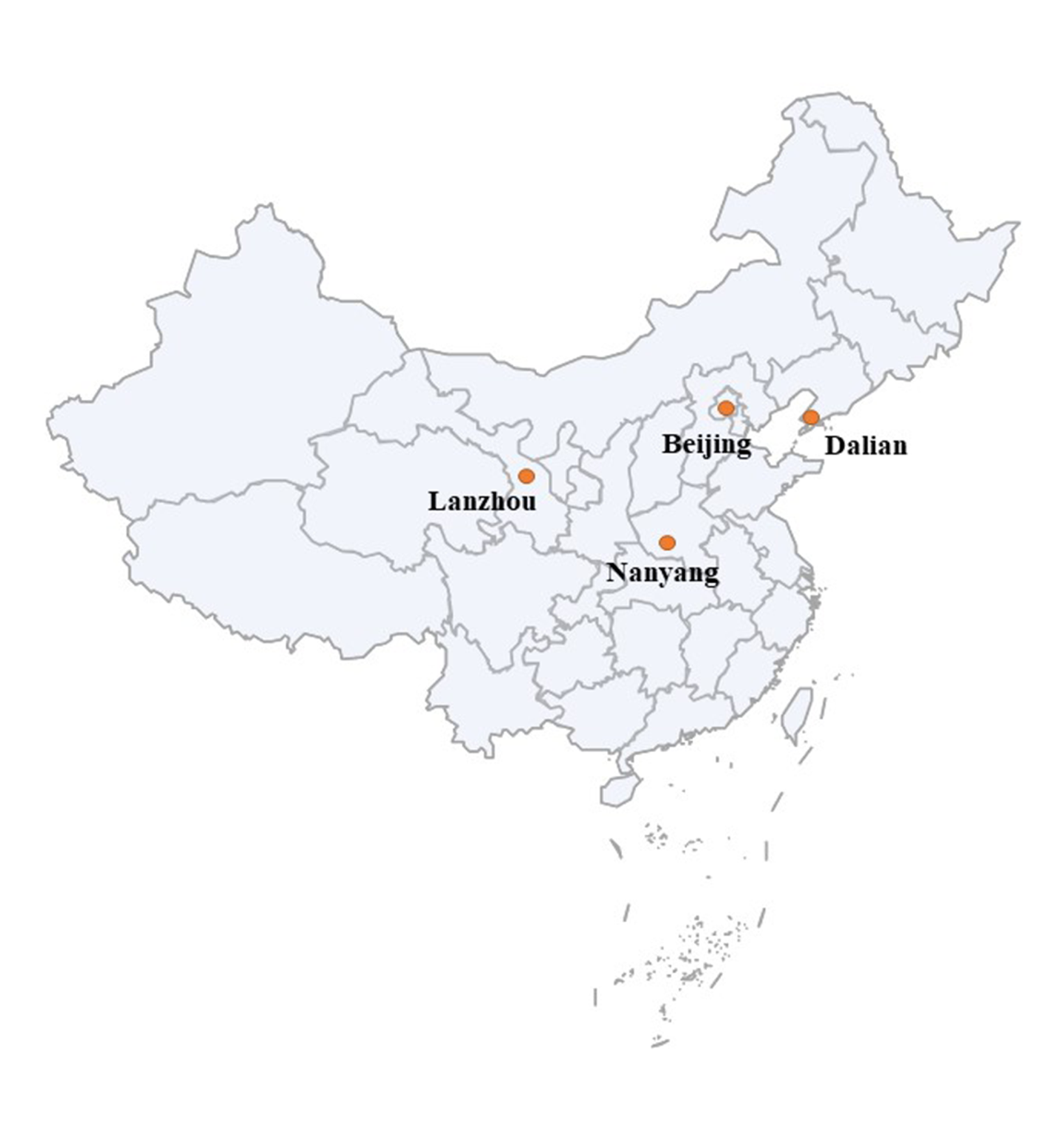

Supplement: Supplementary Figure 1 — Four representative cities in northern China. [file Image_1.TIF]
