# Supplementary material for: Assessing Visceral Obesity and Abdominal Adipose Tissue Distribution in Healthy Populations Based on Computed Tomography: A Large Multicenter Cross-Sectional Study
Source: Front Nutr. 2022 Apr 25;9:871697. doi: 10.3389/fnut.2022.871697 (PMC9082940; doi:10.3389/fnut.2022.871697)
Supplement: Supplementary Table 1 — CT units used and detailed technical parameters. [file Table_1.DOCX]

Supplementary table 1 CT units used and detailed technical parameters

| Hospital | CT units | No. of channels | tube voltage(kV) | Tube current(mA) | detector collimation(mm) | slice thickness(mm) | reconstruction thickness(mm) | pitch(mm) |
| --- | --- | --- | --- | --- | --- | --- | --- | --- |
| Beijing Youan Hospital, Capital Medical University | GE LightSpeed | 64 | 120 | 380 | 0.625 | 5 | 0.625 | 5 |
| the Second Hospital of Dalian Medical University | Philips Ingenuity Core | 64/128 | 120 | automatic modulation | 0.625 | 3 | 0.625 | 0.8 |
| the First Hospital of Lanzhou university | Siemens Somatom Sensation | 64 | 120 | 380 | 0.625 | 5 | 0.625 | 5 |
| Nanyang First People's Hospital | Siemens Somatom Definition Flash  GE Optima CT540 | 128  16 | 140  140 | 800  440 | 0.6  0.625 | 0.6  0.625 | 0.4  0.625 | 0.35-3.2  0.5625-1.75 |

Supplementary table 2. Distribution of body composition by sex and BMI

| Sex | Male (n=902) | | | Female (n=885) | | |
| --- | --- | --- | --- | --- | --- | --- |
| BMI (kg/m^2^) | 18.5–25 (n=630) | 25–30 (n=237) | ≥30(n=35) | 18.5–25 (n=654) | 25–30 (n=197) | ≥30(n=34) |
| Variables |  |  |  |  |  |  |
| L3-VATA, cm^2^ | 97.9 (94.1) | 167.4 (77.4) | 209.6 (87.6) | 53.0 (62.0) | 114.4 (79.4) | 142.2 (71.9) |
| L3-VATI, cm^2^/m^2^ | 31.9 (30.1) | 55.2 (27.6) | 70.1 (28.4) | 20.1 (24.0) | 43.1 (31.6) | 53.9 (27.2) |
| L3-SATA, cm^2^ | 101.8 (57.1) | 152.2 (55.2) | 248.9 (93.3) | 129.8 (68.2) | 195.6 (65.7) | 296.8 (101.8) |
| L3-SATI, cm^2^/m^2^ | 34.0 (17.6) | 50.9 (18.2) | 81.39 (32.4) | 49.7 (25.1) | 76.3 (25.6) | 116.9 (31.3) |
| L3-TATA, cm^2^ | 206.7 (140.1) | 323.9 (111.5) | 437.3 (121.0) | 189.8 (116.4) | 317.2 (116.2) | 459.1 (161.8) |
| L3-TATI, cm^2^/m^2^ | 67.1 (45.4) | 110.1 (35.1) | 148.6 (134.6) | 72.8 (42.7) | 120.4 (45.0) | 176.6 (54.8) |

Data are shown as median (interquartile range).
